# Editorial: Advances on participation perspective in rehabilitation sciences

**DOI:** 10.3389/fresc.2025.1686925

**Published:** 2025-10-01

**Authors:** Mert Doğan, Özgün Kaya Kara

**Affiliations:** Physiotherapy and Rehabilitation Department, Faculty of Health Sciences, Akdeniz University, Antalya, Türkiye

**Keywords:** participation, rehabilitation, ICF, framework, body function and structure, activity

**Editorial on the Research Topic**
Advances on participation perspective in rehabilitation sciences

## 1 Introduction

The International Classification of Functioning, Disability and Health (ICF) defines “participation” as an individual's “involvement in life situations” and identifies it as a primary objective of rehabilitation (1). In this regard, rehabilitation sciences are undergoing a substantial paradigm shift from the traditional biomedical model, which concentrates solely on restoring impaired body structures and functions, to a comprehensive biopsychosocial model. This model emphasizes the individual's meaningful interaction with their environment and their ability to effectively maintain social roles, including personal, social, and occupational roles (2, 3). This conceptual transformation is predicated on the understanding that the effectiveness of rehabilitation outcomes should be assessed not only by improvements in clinical and physiological parameters but, more importantly, by the individual's full, active, and inclusive integration into social life.

The research topic “*Advances on participation perspective in rehabilitation sciences*” was developed to encapsulate the most recent advancements in this field, offering a platform for interdisciplinary perspectives that integrate clinical practice, technological innovations, and policy considerations. Several themes are apparent in the articles contained within this collection. Firstly, innovative strategies for assessing participation are explored, including technology-assisted measurement systems, patient-reported outcome measures, and context-sensitive frameworks. Secondly, interventions aimed at enhancing participation – ranging from community-based programs to individualized therapy approaches – are presented, emphasizing the role of environmental facilitators and the removal of participation barriers. Thirdly, the importance of cultural, socioeconomic, and policy contexts in shaping participation opportunities is emphasized, advocating for a more inclusive and rights-based approach to rehabilitation.

## 2 Overview of the reseach topic

The articles contained within this collection are demonstrative of a broad spectrum of populations, methodologies, and intervention strategies, thus illustrating the multifaceted nature of participation in rehabilitation.

Rozenberg et al. conducted a brief research report that brings together the outcomes of expert meetings and findings from narrative reviews to establish priorities for telerehabilitation in solid organ transplant recipients. Their analysis underscores the promise of remote interventions in sustaining and enhancing participation after transplantation, while also drawing attention to persistent challenges in ensuring equitable access and continuity of care.

In another contribution, Boulay et al. present a case report describing a novel multimodal therapeutic approach aimed at improving locomotor function and participation in a pediatric population. By combining focal vibration therapy with botulinum toxin injections, the authors illustrate an individualized strategy for optimizing functional mobility outcomes.

Camerota et al. explore the integration of focused muscle vibration with neurocognitive rehabilitation within a neuroplasticity-oriented framework. Their case report highlights the potential of this combined approach to facilitate functional recovery and promote greater participation in daily living activities for individuals with spinal cord injury.

Shifting the focus to organizational dynamics, Steensgaard et al. conducted an original study using action research methodology to examine the barriers faced by nursing staff in embedding patient participation into routine practice during spinal cord injury rehabilitation. Their findings reveal that institutional culture and time constraints are critical factors influencing the adoption of participatory care models.

Finally, Kumar Das et al. investigated the relationship between functional independence and community integration among individuals with spinal cord injury in a resource-limited setting. Their work emphasizes the influence of socio-economic and cultural factors on rehabilitation outcomes, underscoring the need for context-specific strategies to enhance participation.

## 3 Conclusion

The articles in this special issue make a meaningful contribution to a deeper understanding of the concept of “participation” in rehabilitation, both from scientific and practical perspectives. They present approaches that are technologically supported, grounded in contextual realities, and applicable in clinical practice. By placing participation at the core of rehabilitation goals, these contributions emphasize that the success of rehabilitation should be evaluated not only through clinical indicators but also by the extent to which individuals can fully and meaningfully engage in their life roles. The perspectives offered within this framework aim to guide future research, strengthen interdisciplinary collaboration, and encourage the development of policies and practices that recognize participation as a fundamental right in rehabilitation.
